# Sense of country: General and specific factors covary with social identification and predict emigration plans

**DOI:** 10.3389/fpsyg.2022.992028

**Published:** 2022-11-03

**Authors:** Aleksandrs Kolesovs

**Affiliations:** Department of Psychology, University of Latvia, Riga, Latvia

**Keywords:** sense of country, bifactor model, belonging, commitment, perceived opportunities, influence, social identification, emigration plans

## Abstract

Theoretical analyses of person–environment interaction describe complex models, addressing different levels of social systems, while models of the sense of community provide a base for transferring views of this interaction to the national level. This paper presents two studies that explored the structure of the sense of country and its relation to emigration plans and social identification. Study 1 involved 1,005 adults from Latvia. The Sense of Country Inventory (SOCI) included influence, perceived opportunities, belonging, and spatiotemporal commitment as the components of this sense. The bifactor model demonstrated the best fit and confirmed the general factor, integrating components of the sense of country, and specific factors, emphasizing its complexity. The validation demonstrated that the general sense of country is the main negative predictor of emigration plans. Study 2 included 247 participants who completed the SOCI and Identification With All Humanity Scale (IWAH). Correlating with national identification, the sense of country negatively predicted emigration plans that reflected the social identity continuity pathway. In turn, a negative relationship between the sense of country and global identification, which positively predicted emigration plans, revealed a social identity gain pathway. Together, the studies present the integrative nature of the sense of country and its links to emigration plans and national and global social identification.

## Introduction

Complex models of person–environment interaction (e.g., Bronfenbrenner and Morris, 2006) reflect the multilevel nature of social systems. Some components of this interaction are well described in the literature, such as generic models of social identity (Tajfel, 1981) and identification (Leach et al., 2008), which present ethnic, national, and other modes. However, broader views of the interaction with the social environment are less developed. Models of the sense of community successfully integrate multiple elements of the perceived social context (McMillan and Chavis, 1986; Pretty et al., 2003; Talò et al., 2014), while the national level remains underinvestigated. In order to close this gap, some studies address the country as a community (Huang et al., 2020; Weiss-Dagan et al., 2021). The development of a specific model of the sense of country can help establish its dimensional structure and quantify trends in changing multicultural societies (Modood, 2020), solving national conflicts (Moura et al., 2021), and issues connected to migration (Gustafson, 2009; Robins, 2022). The two studies described in this paper explored the dimensional structure of the sense of country and its relations to emigration plans and national and global identification.

The dimensionality of perceived social context has been investigated at the community level for the last five decades. McMillan and Chavis (1986) presented four components of the sense of community: membership, influence, fulfillment of needs, and a shared emotional connection. These components are conceptually interrelated. For example, fulfilling needs strengthens membership, and influences links to emotional connections.

Empirical studies have confirmed close correlations among these components and a higher-order factor, representing the sense of community (Peterson et al., 2008; Lardier et al., 2022). Simultaneously, there has been a continuous search for a better structure of the sense of community at different levels of communities (Chipuer and Pretty, 1999; Prezza et al., 2001; Tartaglia, 2006; Jason et al., 2015; Prati et al., 2021), including at the level of the whole country (Huang et al., 2020; Weiss-Dagan et al., 2021). Some models present a higher number of factors by addressing community subgroups (e.g., Prati et al., 2021), while other analyses use a simpler factorial structure (Prezza et al., 2001; Tartaglia, 2006; Weiss-Dagan et al., 2021) that demonstrates the variability of the structure of the sense of community. The tendencies revealed in previous studies are significant for exploring the dimensionality of the sense of country. The four-factor model of the sense of community (McMillan and Chavis, 1986) provides a well-developed basis for modeling the sense of country. Simultaneously, testing alternative factorial models will specify the structure of this sense.

There is empirical evidence for transferring the aforementioned components to the national level. *Membership* reflects a sense of belonging and relatedness, which is also significant at the national level (Dekel and Nuttman-Shwartz, 2009; Hou et al., 2018; Kolesovs, 2021). *Influence* integrates personal and group impacts on the system and its members (McMillan and Chavis, 1986). It includes activation of group membership (Fritsche et al., 2008) and collective agency (Bandura, 2002) in dealing with personal and system-level challenges. The analysis of perceived impacts on the country (Kolesovs et al., 2021) confirms the integration of personal control with perceived control from the mesosystem and the people of the country. *Fulfillment of Needs* addresses the rewarding value of the person–group association. It links to perceived opportunities and constraints on achieving personal goals, which channel individual socialization and lifespan (Nurmi, 2004; Heckhausen and Buchmann, 2019) and predict the association of personal life with the community and country (Pretty et al., 2003; Kolesovs, 2019). These findings provide a reason for refocusing fulfillment of needs onto perceived *Opportunities* for personal goals. *Shared Emotional Connection* refers to shared history, current events, and investment in the future of a community, united with emotional and spiritual bonds with people in the neighborhood. It reflects the complexity of shared connections, including current relationships and views of the past and future, forming continuity in time (Sani et al., 2008; David and Bar-Tal, 2009; Kolesovs, 2021).

The revealed complexity of *Shared Emotional Connection* resulted in a modification of the structure of the sense of country. Studies demonstrate the possibility of analyzing two components of the sense of belonging to the country (Dekel and Tuval-Mashiach, 2012; Kolesovs, 2021). The first represents relational belonging, which reflects the fundamental tendency of membership, belonging, and relatedness (Hagerty et al., 1992; Baumeister and Leary, 1995), while the second involves current, retrospective, and prospective views of commitment to the country. The role of spatiotemporal commitment in predicting emigration (Kolesovs, 2021) points to functional differences between the two components. Therefore, in the current research, *Shared Emotional Connection* was restructured by considering continuity in time as *Spatiotemporal Commitment* to the country and socioemotional bonds with people as part of *Relational Belonging*, integrated with *Membership*.

As a result, the model of the sense of country involved four main components: *Influence*, perceived *Opportunities* for the fulfillment of personal goals, *Relational Belonging*, and *Spatiotemporal Commitment* to the country. Close interrelations of these components are supported in the frame of four processes of socialization—channeling, selection, adjustment, and reflection (Nurmi, 2004). Selected personal goals interact with perceived opportunities for their fulfillment, which channels individual socialization. Adjustment includes attributions of control and influence and resetting goals in the face of change. Reflection forms the sense of belonging and the sense of country in general. Therefore, described socialization processes depict a significant part of person–environment interaction, and their unity allows a general factor of the sense of the country to be hypothesized.

## Study 1

**Figure 1:**
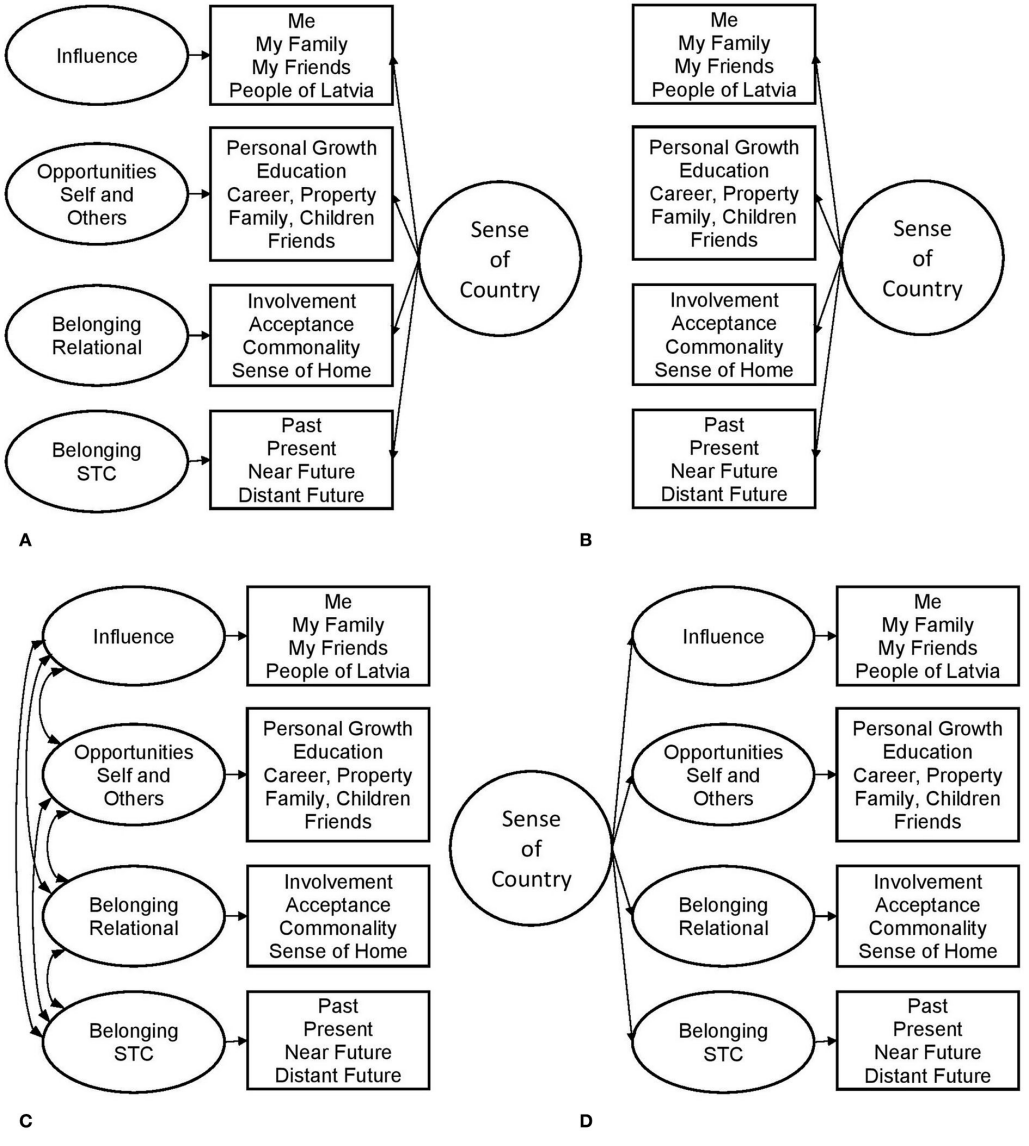
Factorial models of the sense of country: **(A)** bifactor, **(B)** unidimensional, **(C)** correlated factors, and **(D)** higher-order factor. STC, Spatiotemporal Commitment.

This study explored the dimensionality of the sense of country by testing a set of factorial models (Figure 1). The bifactor model (Figure 1A) includes the hypothesized general factor and specific factors linked to different psychological constructs (Chen et al., 2012). Within this model, the general factor accounts for the shared commonality of items, representing the sense of country, while specific factors reflect the unique influence of specific components. For example, perceived influence on the country can depict personal agency, mediated by its collective and proxy modes at the national level (Bandura, 2002). Opportunities for achieving personal goals are related to individual future orientation (Nurmi, 1991; Seginer et al., 2004) and regulatory mechanisms of socialization (Nurmi, 2004), including values as trans-situational goals (Schwartz, 1992; Sagiv et al., 2017). As a result, opportunities can form two subfactors representing self-oriented and other-oriented personal goals (Nurmi, 2004) or self-enhancing and self-transcendent values (Schwartz, 1992). This split should be tested as an alternative to opportunities, joined into a single factor. The relational component of the sense of belonging to the country can associate with components of national identification (Leach et al., 2008), and the spatiotemporal commitment can link to other levels of social systems (Kolesovs, 2019) or individual time perspective (Andre et al., 2018).

Three models formed alternatives to the bifactor model. The unidimensional model (Figure 1B) considered all variances explained by one factor—the sense of country. The correlated factors model (Figure 1C) presented the sense of country as a multidimensional construct, while correlations reflect the variance shared by these dimensions. The sense of country as a higher-order factor, mediated by first-level factors, formed the third model (Figure 1D).

The relationship between components of the sense of country and considering emigration provided the basis for testing the model's validity. Perceived lack of opportunities predicts planning migration from a community (Pretty et al., 2003; Arcidiacono et al., 2007; Kley and Mulder, 2010; Kley, 2017; Wenham, 2020) and is among the reasons for emigration from a country (Prankumar et al., 2021). A lower level of spatiotemporal commitment facilitates the consideration of emigration (Kolesovs, 2021), but the sense of personal influence on a country can strengthen prospective belonging to it (Kolesovs, 2019). Therefore, a negative relationship was expected between the general factor of the sense of country and planning emigration. Simultaneously, personal agency forms a resource required for emigration (Willekens, 2017), and this specific factor can be positively related to emigration plans.

In parallel to validation, exploring associations between the sense of country and emigration plans could facilitate understanding of this problem in Latvia. Emigration contributes significantly to the country's depopulation (Central Statistical Bureau of Latvia, 2021) and is a reason for social programs focused on remigration (Ministry of Finances of the Republic of Latvia, 2021). Studies on communities of emigrants and remigration integrate psychological perspectives, including motivation, belonging, and attachment to the host country and Latvia (Koroleva, 2019; Šupule, 2021). Simultaneously, analyses of emigration trends remain predominantly sociological (e.g., Hazans, 2019). Psychological studies address relatively narrow constructs associated with emigration plans (e.g., Kolesovs, 2021), while young people in Latvia consider emigration a well-established way of dealing with socioeconomic challenges (Kolesovs and Kashirsky, 2014). Therefore, revealing the structure of the sense of country and its role in predicting emigration plans can extend the psychological basis for the person–country interaction and inform relevant programs.

### Materials and methods

#### Participants

A sample of 1,005 adults from Latvia, aged 18 to 75 (*M* = 47.06, *SD* = 14.57 years), represented this population segment in four quotas: gender, age, living place, and ethnicity. Table 1 presents the unweighted demographic characteristics of the sample because of the current focus on the structure of the sense of country.

**Table 1:** Unweighted demographic characteristics of the sample of resident adults from Latvia (*N* = 1,005).

**Characteristic**	**Count, *n* (%)**
**Gender**	
Females	536 (53.3%)
Males	469 (46.7%)
**Age groups**	
18–30	137 (13.6%)
31–40	243 (24.2%)
41–50	203 (20.2%)
51–60	198 (19.7%)
61–70	179 (17.8%)
Over 70	45 (4.5%)
**Living place**	
Riga (the capital)	359 (35.7%)
Another city or town	389 (38.7%)
Rural area	257 (25.6%)
**Ethnic group**	
Latvian	611 (60.8%)
Russian	263 (26.2%)
Polish	35 (3.5%)
Belarusian	26 (2.6%)
Ukrainian	22 (2.2%)
Other	48 (4.7%)
**Ethnolinguistic group**	
Latvian speakers	631 (62.8%)
Russian speakers	343 (34.1%)
Other	31 (3.1%)
**Education**	
Higher	579 (57.6%)
Other	426 (42.4%)
**Employment**	
Employed	749 (74.5%)
Other	256 (25.5%)
**Marital status**	
Married	699 (69.6%)
Not married	306 (30.4%)
**Experience of studying or working abroad**	
Yes	274 (27.2%)
No	731 (72.8%)

The distribution of other demographic characteristics indicates that the sample includes more participants with higher education than the general population (Central Statistical Bureau of Latvia, 2021). It presents some shifts in socioeconomic status.

#### Measures

The Sense of Country Inventory (SOCI) included subscales for testing the model and assessing components of the sense of country. It adapted the assessment of perceived influence from a study on the perceived impact on the country (Kolesovs et al., 2021). Participants used a seven-point scale from “no impact” (1) to “maximal impact” (7) to answer a question: “To what extent do the factors listed below impact Latvia?.” Four items included: *you* (personal impact), *your family members, your friends*, and *the people of Latvia*. Therefore, the subscale presented self-evaluation in contrast to objective measures of a specific component of agency (Moore, 2016).

The next subscale measured perceived opportunities for personal goals in seven domains: *education, career, property, personal growth, family, children*, and *relationships with friends*. Participants used a seven-point scale from “not at all” (0) to “maximum” (6) to answer the question: “Does Latvia provide opportunities for fulfilling your goals in the following domains?.”

Two subscales of the sense of belonging to the country (Kolesovs, 2021) represented relational belonging and spatiotemporal commitment. The measure of relational belonging to the country included four items. For example: “I feel a commonality with the people of Latvia.” Participants used a seven-point scale from “minimally” (1) to “maximally” (7) to rate their answers. The Cronbach's alpha score for this subscale was 0.82, and the test–retest reliability score was 0.78.

The assessment of spatiotemporal commitment included one question: “To what extent do you associate your life with Latvia?.” Participants assessed the level of association in four temporal categories—*the recent past, present, near future*, and *distant future*—on a seven-point scale (from “minimally” to “maximally”). For this subscale, the Cronbach's alpha score was 0.85, and the test–retest reliability score was 0.81.

In addition to the SOCI, the assessment of emigration plans included four items (Kolesovs, 2021): “I am looking for information on emigration opportunities,” “I have chosen the country I will go to live in,” “I have a clear emigration plan,” and “I am fulfilling my emigration plan step by step.” Participants used a seven-point scale, anchored by “completely disagree” (1) and “completely agree” (7) to indicate their agreement with these statements. The Cronbach's alpha score for this scale was 0.91. As demonstrated in a previous study (Kolesovs, 2021), residuals of having plans and their fulfillment correlated.

#### Procedure

The research project was supported by the University of Latvia and received the approval of the Research Ethics Committee of the Institute of Cardiology and Regenerative Medicine of the University of Latvia, No 125/2020. The SOCI and questions regarding emigration were shared with colleagues in the frame of scientific cooperation. Data collection occurred through a web omnibus interview of resident adults in Latvia from September 10 to 14, 2020; and data regarding the SOCI and emigration plans became available.

Calculations of the minimum *a priori* sample size (Soper, 2022) resulted in sample sizes of 207–236 participants for 19 observed indicators, four to six latent variables (for different versions of the model), an anticipated minimal effect size of 0.30 (Kolesovs, 2021), an alpha level of 0.05, and a power of 0.95. The subsamples presenting participants' demographic characteristics satisfied this requirement in testing the invariance of the model.

IBM SPSS Statistics for Windows 22.0 (RRID:SCR_019096) was applied for regular statistical tests. Four packages for R Project for Statistical Computing (RRID:SCR_001905) were applied for specific purposes. Confirmatory factor analysis and structural equation modeling were conducted using “lavaan” 0.6–11 (Rosseel, 2012). The package “psych” 2.2.5 (Revelle, 2022) provided calculations of hierarchical reliability coefficients. The “BifactorIndicesCalculator” 0.2.2 package (Dueber, 2021) calculated the bifactor model's indices described in Rodriguez et al. (2016). Model invariance was established using “semTools” 0.5–6 (Jorgensen et al., 2022). Testing invariance focused on the metric equivalence of the model between subgroups (Putnick and Bornstein, 2016).

### Results

#### Factorial structure

Table 2 presents the fit of factorial models of the sense of country. Bifactor Model 1 included perceived opportunities as one specific factor. All items loaded positively on the general factor (from 0.64 to 0.89). Simultaneously, loadings of opportunities for self-related goals on a specific factor were positive (from 0.15 to 0.21), and those of others-related goals were negative (from −0.31 to −0.44). Model 2 included these items as two separate factors—*Self-Related Opportunities* and *Others-Related Opportunities*. The model fit increased, robust ΔCFI = 0.013, ΔRMSEA = −0.011, and loadings on each specific factor were positive. Therefore, the bifactor model with five specific factors demonstrated the best fit and provided the baseline for further comparisons.

**Table 2:** Fit indices of factorial models of the sense of country (*N* = 1,005).

**Model**	**χ^2^**	***df***	**CFI**	**TLI**	**RMSEA [90% CI]**	***p_*RMSEA*_***	**SRMR**	**AIC**	**SCF**
1	601.78	133	0.952	0.939	0.059 [0.055, 0.063]	0.000	0.079	60577.60	1.43
2	469.69	133	0.966	0.956	0.050 [0.046, 0.054]	0.462	0.056	60385.10	1.42
3	4791.03	152	0.529	0.470	0.174 [0.171, 0.178]	0.000	0.131	66565.95	1.44
4	579.29	142	0.956	0.947	0.055 [0.052, 0.059]	0.011	0.057	60547.54	1.47
5	704.23	147	0.943	0.934	0.061 [0.058, 0.065]	0.000	0.075	60712.46	1.45

Model 1, Bifactor with four specific factors; Model 2, Bifactor with five specific factors; Model 3, Unidimensional; Model 4, Five correlated factors; Model 5, Higher-order factor; SCF, Scaling correction factor for Satorra–Bentler correction. All chi-square tests were significant, *p* < 0.001.

Unidimensional Model 3 demonstrated the worst fit, confirming the complexity of the sense of country. Five correlated factors of Model 4 and a higher-order factor of Model 5 also did not improve the fit of Model 2. As a result, bifactor Model 2 outperformed Model 4 as its best alternative with robust ΔCFI = 0.011 and ΔRMSEA = −0.007.

#### Reliability and statistical indices

Table 3 presents factor loadings, statistical indices, and reliability coefficients of the bifactor model. Loadings on the general factor—*Sense of Country*—varied from 0.37 to 0.79 and were significant (*p* < 0.001). All loadings on specific factors also were significant except for perceived property opportunities, which were loaded only on the general factor. Percentage of reliable variance (PRV) and a hierarchical McDonald's omega coefficient higher than 0.80 supported the reliability of the general factor (Reise et al., 2013). Among the specific factors, *Influence* demonstrated substantial variance of a specific construct (PRV = 0.76, ω_HS_ = 0.70), which can be analyzed in parallel with the general factor. In addition, *Spatiotemporal Commitment* indicated the potential presence of such a construct (PRV = 0.52, ω_HS_ = 0.47). For the other specific factors, PRV values were lower than 0.50, and the general factor predominantly explained their variance (Reise et al., 2013). Factor determination scores exceeding 0.90 and construct replicability over 0.70 (Rodriguez et al., 2016) confirmed the significance of the general factor, *Influence*, and *Spatiotemporal Commitment* in the model.

**Table 3:** Factor loadings, statistical indices, and reliability of the bifactor model of the sense of country (*N* = 1005).

**Items**	**SOC-G**	**INFL**	**SRO**	**ORO**	**RB**	**STC**	***h* ^2^**	**Relative Bias**
INFL Personal	0.38	0.83					0.85	0.17
INFL Family members	0.37	0.87					0.90	0.19
INFL Friends	0.43	0.80					0.82	0.13
INFL People of Latvia	0.50	0.50					0.49	0.06
SRO Growth	0.76		0.49				0.82	0.05
SRO Education	0.69		0.55				0.78	0.08
SRO Work	0.77		0.47				0.82	0.03
SRO Property	0.79		0.09^a^				0.64	0.05
ORO Family	0.61			0.56			0.67	0.01
ORO Children	0.58			0.59			0.68	0.02
ORO Friends	0.66			0.36			0.56	0.02
RB Commonality	0.60				0.62		0.75	0.07
RB Involvement	0.57				0.39		0.48	0.04
RB Acceptance	0.70				0.42		0.67	0.08
RB Home	0.51				0.41		0.43	0.08
STC Past	0.50					0.44	0.45	0.04
STC Present	0.57					0.65	0.75	0.03
STC Near future	0.61					0.79	0.99	0.11
STC Distant future	0.61					0.54	0.67	0.32
*M (SD)*	4.96 (1.12)	3.25 (1.60)	5.16 (1.63)	5.64 (1.51)	4.63 (1.52)	6.27 (1.14)		0.08 (0.08)
ω	0.96	0.92	0.92	0.84	0.84	0.90		
ω_H_/ω_HS_	0.81	0.70	0.20	0.33	0.31	0.47		
PRV	0.84	0.76	0.22	0.39	0.37	0.52		
FD	0.93	0.96	0.78	0.78	0.79	0.96		
H	0.93	0.88	0.51	0.53	0.55	0.75		
ECV	0.52							
PUC	0.84							

Coefficients for empty cells were not calculated. SOC-G, Sense of Country (General Factor); INFL, Influence; SRO, Self-Related Opportunities; ORO, Others-Related Opportunities; RB, Relational Belonging; STC, Spatiotemporal Commitment; ω_H_, Hierarchical ω for the general factor; ω_HS_, Hierarchical ω for specific factors; PRV, Percentage of reliable variance; FD, Factor determinacy; H, Construct replicability; ECV, Explained common variance; PUC, Percentage of uncontaminated correlations. ^a^Nonsignificant factor loading (other loadings were significant at *p* < 0.001).

The explained common variance of the general factor was 0.52, indicating that the percentage of common variance attributable to it was higher than half of the common variance. The average relative bias of the whole scale was moderate, and relative biases of three items of *Influence* and two of *Spatiotemporal Commitment* were strong, following cut-offs of 0.05 for moderate and 0.10 for strong biases (Bader et al., 2022). These findings also confirm the presence of the leading general factor and a non-trivial role of specific factors.

#### Model invariance

Focusing on the dimensionality of the sense of country required establishing the metric invariance of the model. Equivalence of factor loading was explored for gender, age, living place, higher education, occupation, marital status, and the ethnolinguistic group. The model demonstrated full metric invariance regarding participants' gender, $\Delta \chi_{\left(32\right)}^{2}$ = 38.88, *p* = 0.188, ΔCFI = 0.001, ΔRMSEA = 0.003; higher education, $\Delta \chi_{\left(32\right)}^{2}$ = 40.58, *p* = 0.142, ΔCFI = 0.001, ΔRMSEA = 0.002; and marital status, $\Delta \chi_{\left(32\right)}^{2}$ = 36.97, *p* = 0.250, ΔCFI = 0.000, ΔRMSEA = 0.003.

The following comparisons revealed the partial metric invariance of the model. It was established for the living place (Riga vs. other places), $\Delta \chi_{\left(31\right)}^{2}$ = 42.10, *p* = 0.088, ΔCFI = 0.001, ΔRMSEA = 0.002. The loading of the perceived impact of the people of Latvia on *Influence* was slightly lower in the capital, 0.42, than in the rest of Latvia, 0.53, $\Delta \chi_{\left(1\right)}^{2}$ = 4.67, *p* = 0.031. For Latvian and non-Latvian speakers, the model also was partially invariant after relaxing loadings of opportunities for relationships with friends and perceived acceptance on corresponding specific factors, $\Delta \chi_{\left(30\right)}^{2}$ = 40.46, *p* = 0.096, ΔCFI = 0.001, ΔRMSEA = 0.002. Factor loading of opportunities for relationships was lower for Latvian speakers, 0.24, than for non-Latvian speakers, 0.45, $\Delta \chi_{\left(1\right)}^{2}$ = 5.79, *p* = 0.016. Similarly, the loading of acceptance was lower for Latvian, 0.37, than for non-Latvian, 0.58, speakers, $\Delta \chi_{\left(1\right)}^{2}$ = 14.13, *p* < 0.001.

Associated with decreasing mobility plans in Latvian adults (Hazans, 2019), age 40 formed the split-point for establishing invariance in age groups. The results demonstrated the partial metric invariance of the model, $\Delta \chi_{\left(30\right)}^{2}$ = 42.59, *p* = 0.063, ΔCFI = 0.001, ΔRMSEA = 0.002. The factor loading of opportunities for relationships with friends on *Others-Related Opportunities* was higher in older, 0.45, than in younger, 0.20, adults, $\Delta \chi_{\left(1\right)}^{2}$ = 6.60, *p* = 0.010. Conversely, the factor load of the sense of commonality on *Relational Belonging* was higher in younger, 0.68, than in older, 0.57, participants, $\Delta \chi_{\left(1\right)}^{2}$ = 4.66, *p* = 0.031.

Testing invariance regarding participants' employment also revealed the partial metric invariance of the model, $\Delta \chi_{\left(28\right)}^{2}$ = 37.86, *p* = 0.101, ΔCFI = 0.001, ΔRMSEA = 0.002. The factor load of personal growth on *Self-Related Opportunities* was lower in employed, 0.48, than in non-employed, 0.57, participants, $\Delta \chi_{\left(1\right)}^{2}$ = 4.73, *p* = 0.030. Similarly, the load of opportunities for relationships with friends on *Others-Related Opportunities* was lower in employed, 0.32, than in non-employed, 0.51, respondents, $\Delta \chi_{\left(1\right)}^{2}$ = 3.85, *p* = 0.049. Participants' employment impacted loadings of two items on the general factor. Loading of personal impact was higher in employed, 0.40, than in non-employed, 0.32, respondents, $\Delta \chi_{\left(1\right)}^{2}$ = 9.25, *p* = 0.002, while loading of commitment to the country in the present was higher in non-employed respondents, 0.66 vs. 0.57, $\Delta \chi_{\left(1\right)}^{2}$ = 13.95, *p* < 0.001.

#### Predicting emigration plans

**Figure 2:**
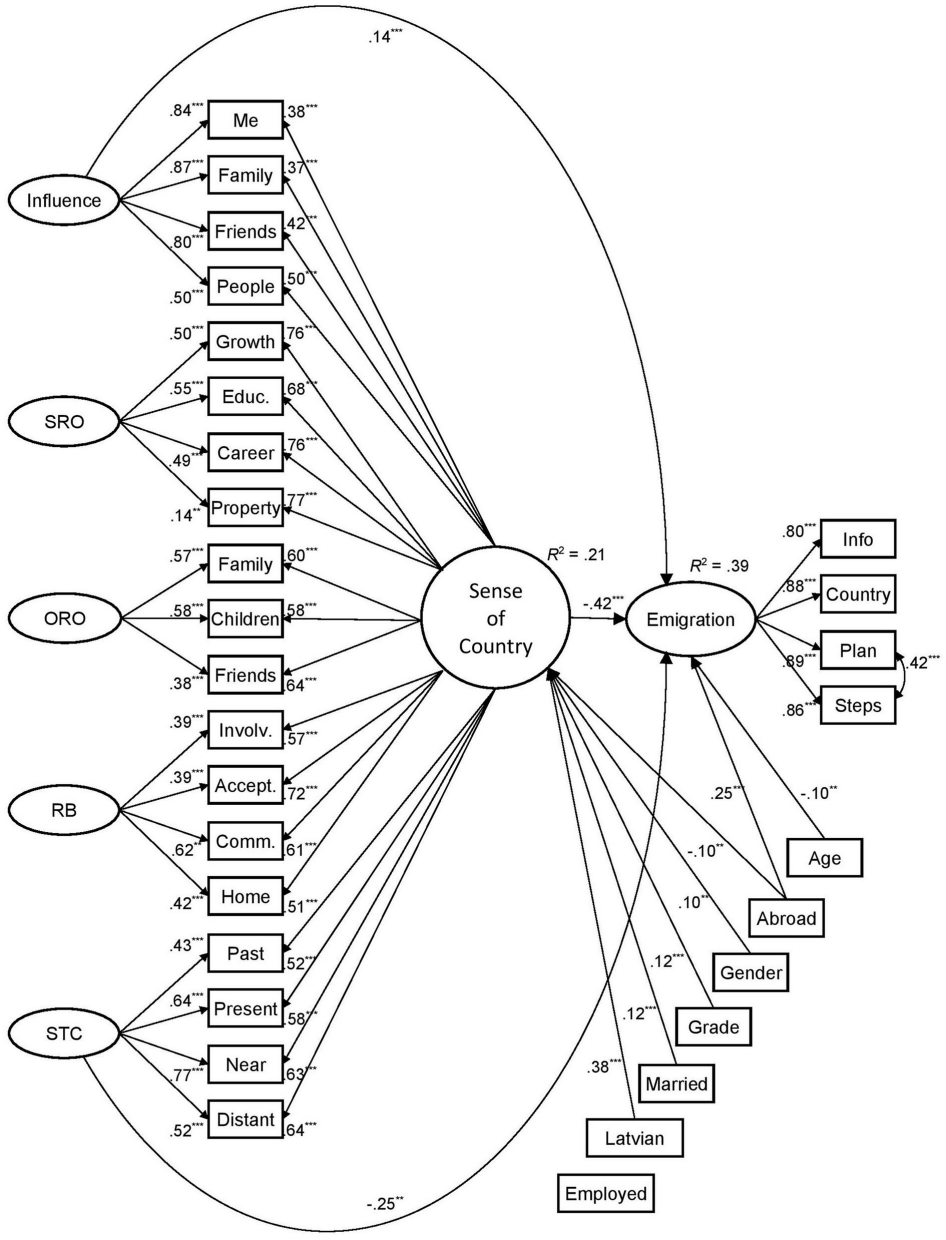
The general and specific factors of the sense of country and demographic variables predicting emigration plans in adults from Latvia (*N* = 1,005; SRO, Self-Related Opportunities; ORO, Others-Related Opportunities; RB, Relational Belonging; STC, Spatiotemporal Commitment. Standardized loadings, covariances, and significant regression coefficients are presented. ***p < 0.001, **p < 0.01).

Testing the bifactor model for predicting emigration plans involved demographic variables and participants' experience of studying or working abroad (Figure 2). Psychometric properties of the scale presenting emigration plans demonstrated its high reliability (Table 4). The predictive model explained 39% of the variance of planning emigration. Its fit was acceptable: $\chi_{\left(351\right)}^{2}$ = 1409.09, *p* < 0.001, CFI = 0.925, TLI = 0.911, RMSEA = 0.055 with 95% CI [0.052, 0.057], *p*_*RMSEA*_ = 0.002, and SRMR = 0.061.

**Table 4:** Psychometric properties of the emigration plan scale (*N* = 1005).

**Scale and items**	***M (SD)***	**Item-total correlation/*M (SD)***	**α/α if item dropped**
Emigration plan (Total score)	1.65 (1.27)	0.82 (0.05)	0.92
Item 1: Looking for information	1.91 (1.66)	0.75	0.92
Item 2: Country chosen	1.70 (1.52)	0.83	0.88
Item 3: Emigration plan	1.50 (1.24)	0.86	0.88
Item 4: Fulfilling the plan	1.47 (1.22)	0.85	0.88

All corrected item-total correlations were significant at *p* < 0.001.

The results showed that a lower level of the sense of country is the main predictor of considering emigration. Among the specific factors, *Influence* positively predicted considering emigration, while *Spatiotemporal Commitment* was a negative predictor. *Relational Belonging* and *Self*- and *Others-Related Opportunities* did not contribute to the prediction. These findings align with the reliability of specific factors, indicating the presence of independent constructs for *Influence* and *Spatiotemporal Commitment*.

Two demographic variables were predictors for considering emigration. Emigration plans decreased with age and were higher in adults experienced in studying or working abroad. Simultaneously, the effects of demographics were more visible in the general sense of country. It was higher in Latvian speakers, female, married, and graduated participants. Participants who experienced studying or working abroad demonstrated a lower sense of country. In sum, demographic variables added 5% to the explained variance of emigration plans.

### Discussion

The results identified the bifactor model as the best representation of the explored construct. The general factor reflects the sense of country, which integrates belonging, commitment, perceived opportunities for self-related and others-related personal goals, and influence. These findings align with the view of intertwined psychological processes of socialization (Nurmi, 2004). In parallel, influence and spatiotemporal commitment reflected additional psychological constructs that emphasize complex relationships among components of the sense of country.

Splitting perceived opportunities into specific factors—*Self-Related Opportunities* and *Others-Related Opportunities*—indicated the significance of both groups of developmental tasks in channeling individual socialization (Nurmi, 2004) and striving toward self-oriented and other-oriented values (Schwartz, 1992). Compared with the model with one polarized specific factor, the model with two specific factors emphasizes that individuals consider opportunities for both groups of goals and values as parallel rather than mutually exclusive. Exploring the reliability of both factors confirmed their association with the general sense of country and did not provide evidence for concurrent independent constructs. It stresses the centrality of perceived opportunities in forming the sense of country. Subsequent analysis of emigration plans also confirmed the significance of perceived opportunities in planning personal life and mobility (Pretty et al., 2003; Kolesovs, 2019).

In contrast to opportunities, *Influence* reflects an interaction of the general and a concurrent factor. As a component of the sense of country, it presents a perceived impact on the country and is among the predictors for not considering emigration. Perceived influence integrates with other factors of the sense of community (Peterson et al., 2008; Lardier et al., 2022) and is among the predictors of belonging to the country (Kolesovs, 2019). In parallel, it can present personal agency as a specific component, forming a resource for life changes, including emigration (Willekens, 2017). This assumption is in line with higher item biases for personal influence and the influence of family and friends. It explains revealed inconsistency between a negative prediction of emigration plans by the sense of country and a positive one by a specific component of *Influence*.

*Spatiotemporal Commitment* also included a concurrent psychological construct. In predicting emigration, the effect of a specific component was concordant with the general factor's effect and confirmed its significance in considering emigration (Kolesovs, 2021). The specific factor can represent the continuity of belonging in time at a different level of social system. For example, individual prospects regarding belonging at the level of community predict those at the level of country (Kolesovs, 2019). It can also depict commitment to a physical place (Arcidiacono et al., 2007; Wenham, 2020). Simultaneously, higher item biases indicate an independent contribution of individual future views associated with a future orientation (Nurmi, 1991; Seginer et al., 2004; Andre et al., 2018).

The general factor predominantly explained the variance of *Relational Belonging*. It indicates the significance of belonging in forming the sense of country, previously established in studies on the sense of community (McMillan and Chavis, 1986; Peterson et al., 2008).

In addition to psychological variables, demographic ones play a role in modulating the sense of country. Its association with marriage and graduation indicates involvement in social relationships and fulfillment of personal goals in the domains of family and education (Nurmi, 1991, 2004; Seginer et al., 2004) and a higher social class (Gustafson, 2009). Latvian speakers (the majority group) displayed a higher level of the sense of country. This concurs with findings on the sense of belonging to Latvia in Latvian and Russian (the minority group) speakers (Kolesovs, 2019, 2021).

Experience of working or studying abroad predicted a lower level of the sense of country and a higher level of considering emigration. This can be explained by the experience of mobility (Gustafson, 2009) and a discontinuity in commitment, which predicts considering emigration (Kolesovs, 2021). In contrast to the experience of mobility, age has an inhibitory effect on emigration plans. This effect can represent a longer commitment to a place (Tartaglia, 2006) and a decreasing sense of personal agency in older adults (Moore, 2016).

The present study demonstrates that the female gender predicts a higher sense of country, but the strength of this relationship is relatively low. Similarly, studies on the sense of community revealed slight, nuanced differences (Pretty et al., 2003) or no gender-related effects (Peterson et al., 2008). Therefore, a more detailed investigation of specific components of the sense of country is needed. Considering inconsistent findings on relationships between gender and belonging to the country (Gustafson, 2009; Kolesovs, 2021), these differences could appear in the belonging domain or pragmatic domains of perceived opportunities and influence.

Another topic for further research is the overlap between the sense of country and social identification at the national level. The sense of belonging at the national level constitutes the sense of country and simultaneously forms a component of social identification (Leach et al., 2008). The current analysis does not reveal the relationships between these constructs, and the next step will address this question in the context of planning emigration.

## Study 2

Developing the model of the sense of country raised a question about its relationships with national identification. On the one hand, perceived commonality and involvement in a social system are the basis for belonging (Hagerty et al., 1992), while belonging, solidarity, and in-group bonds and concerns constitute a significant part of social identification at different levels of social systems (Leach et al., 2008; McFarland et al., 2012; Hamer et al., 2021). These components also appear in descriptions of the sense of community in qualitative inquiries (e.g., Bahl et al., 2021). On the other hand, the sense of country integrates belonging with perceived opportunities for achieving personal goals and influence on the country, which do not constitute social identification directly. The overarching construct of socialization (Nurmi, 2004) ensures the unity of these processes. In turn, social identification involves self-stereotyping and perceived in-group homogeneity (Leach et al., 2008), which are not the direct determinants of the sense of country. Therefore, national identification and the sense of country are conceptually overlapping constructs, but each has unique elements. The mentioned overlap and uniqueness can be revealed in the frame of structural analysis.

The Social Identity Model of Identity Change (SIMIC) (Haslam et al., 2021) provides the framework for further analysis of another finding of Study 1—the link between the sense of country and considering emigration. SIMIC describes two modes of individual adjustment to life challenges, including migration. The social identity continuity pathway means maintaining group membership, while the social identity gain pathway means acquiring new membership. Study 1 showed that the general sense of country and its specific component (*Spatiotemporal Commitment*) negatively predict exploration of opportunities and commitment to emigration plans. Therefore, continuity of commitment to the homeland relates to no change in belonging, but its discontinuity relates to gaining new membership. An explicit measure of national identification can specify this trend.

The current study involved Identification With All Humanity (IWAH) (McFarland et al., 2012) to explore identification with new groups. IWAH represents the maximal possible group for identification representing close ties with and concerns about any human being regardless of nationality, religion, or race (McFarland et al., 2013). This identification helps transcend national borders and facilitates international cooperation (Buchan et al., 2011, 2017). Therefore, developing a broader identity can be a mechanism of adjustment to planned emigration, presenting a social identity gain pathway.

The relationships between different levels of identification and belonging are not trivial. Additive and conjunctive strategies for addressing the identities (Buchan et al., 2017) result in an extension of identification to a superordinate group or restricting such extension, respectively. Moreover, Gustafson (2009) demonstrated that the sense of belonging depends on the level of social systems and type of mobility (from daily to international). It means that the prediction of planning emigration by the sense of country should involve national and global identification, and findings will be relevant only for this kind of mobility.

Following Study 1, the current model involved the sense of country and national and global identification as predictors of emigration plans. The sense of country included five components identified in Study 1. Studies on IWAH framed the presentation of the national and global identifications as involving two components—*Bond* and *Concern* (Hamer et al., 2021). In addition, specific factors of *Influence* and *Spatiotemporal Commitment* can increase the prediction of emigration plans.

The model's final composition depends on the relationship between the sense of country and national identification. Three factorial models were suggested for exploring their interaction. The first model hypothesized one general factor for all components of the sense of country and national identification. Correlated general factors of the sense of country and national identification formed the second model. The last one involved two correlated general factors and a link between the two closest specific components—*Relational Belonging* to the country and *Bonds* with the national population.

### Method

#### Participants

The convenience sample consisted of 247 participants from universities in Latvia. Participants were aged 18 to 54 (mean age = 28.80 years, *SD* = 9.34). Table 5 presents detailed characteristics of the sample. It should also be noted that students constituted 96% of the sample.

**Table 5:** Demographic characteristics of the convenience sample of adults (*N* = 247).

**Characteristic**	**Count, *n* (%)**
**Gender**	
Females	195 (79.0%)
Males	47 (19.0%)
No answer	5 (2.0%)
**Age groups**	
18–30	151 (61.1%)
31–40	58 (23.5%)
41–50	35 (14.2%)
51–60	3 (1.2%)
**Ethnic group**	
Latvian	186 (75.3%)
Russian	13 (5.3%)
Another ethnic group	7 (2.8%)
Multiethnic identification	41 (16.6%)
**Ethnolinguistic group**	
Latvian speakers	205 (83.0%)
Russian speakers	40 (16.2%)
Other	2 (0.8%)
**Education**	
Higher	113 (45.7%)
Other	134 (54.3%)
**Employment**	
Employed	150 (60.7%)
Other	97 (39.3%)
**Experience of studying or working abroad**	
Yes	98 (39.7%)
No	149 (60.3%)

#### Measures

Measurement of the sense of country included the SOCI with five subfactors, as established in Study 1. The assessment of emigration plans also included the subscale from Study 1. The Identification With All Humanity Scale (McFarland et al., 2012) was used for measuring social identification. Following Hamer et al. (2021), eight of nine items represented *Bond* and *Concern* as components of identification with a social group. The *Bond* subscale contains four items. For example: “How close do you feel to each of the following groups?.” The *Concern* subscale also includes four items. For example: “When they are in need, how much do you want to help…” Participants used a five-point scale from “not at all” (1) to “very much” (5) to rate their answers. Some items had specific anchors (Hamer et al., 2021). Each item involved three groups—community, national group, and all humanity—for rating answers.

The current study omitted the community level and included global and national levels of identification to reveal links between the sense of country, two levels of identification, and emigration plans. Following Dunwoody and McFarland (2018), the mean of each IWAH item was regressed onto the mean of identification with national items, and the standardized residual formed the “pure” IWAH measure. The cross-cultural comparison (Hamer et al., 2021) revealed Cronbach's alphas from 0.70 to 0.87 for *Bond*, 0.68 to 0.87 for *Concern*, and 0.75 to 0.90 for the total “pure” scores of IWAH. Data on national identification are less detailed. Studies (McFarland et al., 2012; Hamer et al., 2018) presented Cronbach's alphas from 0.70 to 0.85 for its total score. Furthermore, Hamer et al. (2021) preferred a higher-order factorial model to interpret both subfactors of social identification with significant positive loadings. Simultaneously, a bifactor model of identification was among possible representations of the construct. The current study applied the bifactor model to the national and global identification because of the primary focus on the general factors of the psychological constructs under consideration.

#### Procedure

The study was conducted as an extension of the previous project of the University of Latvia. IWAH was translated into Latvian using a back-translation procedure and applied after the authors' feedback on translated versions. Data collection occurred during the fall semester of 2021. Participation in the study was voluntary and anonymous. After confirming informed consent, participants filled in an online inventory in Latvian without a time limit.

Calculations (Soper, 2022) revealed a minimum sample size of 213 participants for 35 observed indicators, 12 latent variables, an anticipated effect size of 0.34 (Study 1), an alpha level of 0.05, and a power of 0.95. The convenience sample satisfied this requirement.

Regular statistical tests were conducted using IBM SPSS Statistics for Windows 22.0 (RRID:SCR_019096). The two packages for R Project for Statistical Computing (RRID:SCR_001905) were used for specific analyses: “lavaan” 0.6–11 (Rosseel, 2012) was applied for confirmatory factor analysis and structural equation modeling; and a comparison of factorial models occurred using “semTools” 0.5–6 (Jorgensen et al., 2022).

### Results

#### Psychometric properties of the IWAH scale

Testing reliability of the IWAH scale (Table 6) confirmed its internal consistency at a range revealed in adaptations in other cultures (Hamer et al., 2021). The Cronbach's alpha coefficients for the total scales were no lower than 0.80. All subscales also demonstrated an acceptable level of internal consistency except for “pure” scores of *Bond* with all humanity, which was slightly under 0.70. Item difficulty and correlations with any level of summary scores confirmed their successful functioning within the scale and subscales.

**Table 6:** Psychometric properties of the IWAH scale (*N* = 247).

**Scales and items**	***M (SD)***	**Item-total correlation/*M (SD)*, subscale**	**Item-total correlation/*M (SD)*, total**	**α/α if item dropped, subscale**	**α/α if item dropped, total**
IWAH (Total score)	3.21 (0.71)		0.53 (0.05)		0.82
Bond	2.84 (0.81)	0.54 (0.07)		0.74	
Item 1	3.21 (1.03)	0.56	0.54	0.67	0.79
Item 2	2.99 (1.22)	0.57	0.61	0.66	0.78
Item 3	3.19 (1.01)	0.58	0.54	0.66	0.79
Item 4	1.96 (1.06)	0.44	0.46	0.74	0.80
Concern	3.59 (0.79)	0.53 (0.05)		0.74	
Item 6	3.42 (1.08)	0.59	0.58	0.64	0.79
Item 7	3.77 (1.10)	0.49	0.53	0.70	0.79
Item 8	3.67 (0.99)	0.49	0.50	0.70	0.80
Item 9	3.49 (1.07)	0.54	0.51	0.67	0.80
“Pure” IWAH (Total score)	0.00 (0.65)		0.51 (0.08)		0.80
Bond	0.00 (0.74)	0.52 (0.10)		0.73	
Item 1	-^a^	0.56	0.57	0.64	0.77
Item 2	-^a^	0.56	0.59	0.65	0.77
Item 3	-^a^	0.60	0.56	0.62	0.77
Item 4	-^a^	0.37	0.39	0.75	0.80
Concern	0.00 (0.72)	0.48 (0.05)		0.69	
Item 6	-^a^	0.54	0.57	0.59	0.77
Item 7	-^a^	0.49	0.53	0.62	0.78
Item 8	-^a^	0.47	0.49	0.63	0.79
Item 9	-^a^	0.41	0.40	0.67	0.80
IWPL (Total score)	3.45 (0.74)		0.60 (0.05)		0.86
Bond	3.14 (0.87)	0.63 (0.06)		0.81	
Item 1	3.51 (0.99)	0.69	0.62	0.74	0.84
Item 2	3.33 (1.23)	0.66	0.68	0.75	0.83
Item 3	3.51 (0.99)	0.63	0.63	0.76	0.84
Item 4	2.20 (1.15)	0.55	0.61	0.80	0.84
Concern	3.76 (0.77)	0.57 (0.05)		0.77	
Item 6	3.75 (0.99)	0.58	0.57	0.71	0.84
Item 7	3.82 (1.07)	0.56	0.56	0.72	0.84
Item 8	3.72 (0.98)	0.51	0.53	0.74	0.85
Item 9	3.74 (0.95)	0.63	0.59	0.68	0.84

IWAH, Identification With All Humanity; IWPL, Identification With People of Latvia; Coefficients for empty cells were not calculated. All corrected item-total correlations were significant at *p* < 0.001. ^a^Means and standard deviations for “pure” IWAH items were 0.00 and 1.00, respectively.

#### Sense of country and national identification

Testing a series of models answered the question about the relationships between the sense of country and social identification at the national level (Table 7). Model 1 reflected one general factor in a bifactor model, integrating five components of the sense of country and two components of national identification. Model 2 involved correlated bifactor models of the sense of country and national identification. This model achieved a slightly better fit to data than Model 1, robust ΔCFI = 0.005, ΔRMSEA = −0.002. Model 3 included the covariance between *Bond* and *Relational Belonging*. It accounted for a specific factor, shared by the sense of country and social identification. This model demonstrated the best fit and significantly improved Model 2, robust ΔCFI = 0.011, ΔRMSEA = −0.005.

**Table 7:** Fit indices of factorial models of relationships between the sense of country and national identification (*N* = 247).

**Model**	**χ^2^**	***df***	**CFI**	**TLI**	**RMSEA [90% CI]**	***p*_*RMSEA*_**	**SRMR**	**AIC**	**SCF**
1	505.08	297	0.927	0.913	0.053 [0.046, 0.060]	0.224	0.060	20052.49	1.23
2	490.22	296	0.932	0.919	0.052 [0.044, 0.059]	0.357	0.058	20031.81	1.22
3	456.43	295	0.943	0.932	0.047 [0.039, 0.055]	0.732	0.055	19995.89	1.23

Model 1, Bifactor with one general factor; Model 2, Bifactor with two general factors; Model 3, Model 2 with covarying *Relational Belonging* and *Bond* specific components. SCF, Scaling correction factor (Satorra–Bentler correction). All chi-square tests were significant, *p* < 0.001.

#### Sense of country, social identification, and emigration plans

**Figure 3:**
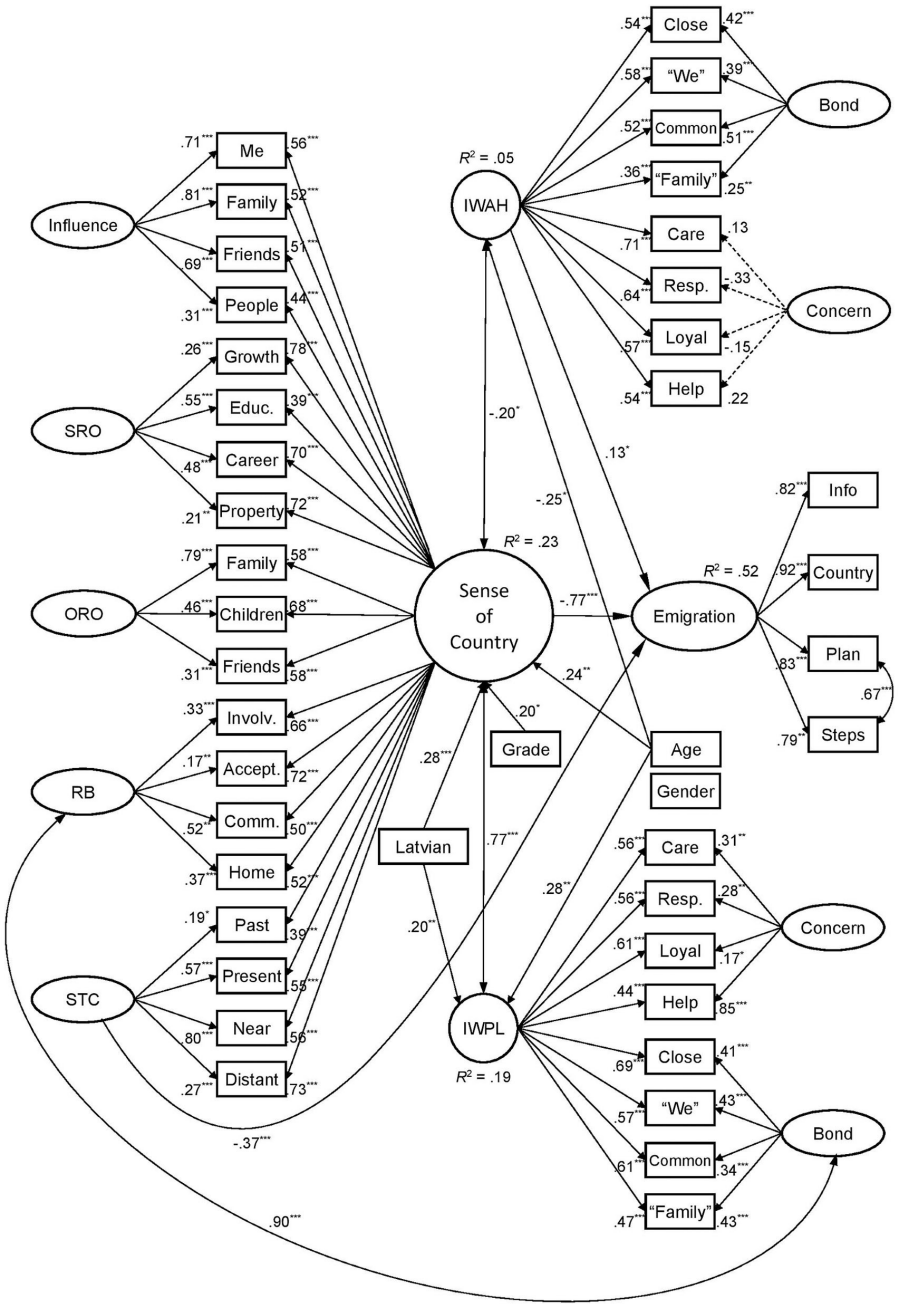
Predicting emigration plans by the sense of country, national and global identification, and demographic variables (*N* = 242; SRO, Self-Related Opportunities; ORO, Others-Related Opportunities; RB, Relational Belonging; STC, Spatiotemporal Commitment; IWAH, Identification With All Humanity; IWPL, Identification With People of Latvia. Dashed lines depict nonsignificant factor loadings. Standardized loadings, covariances, and significant regression coefficients are presented. ***p < 0.001, **p < 0.01, *p < 0.05).

The next step tested the sense of country, identification with the people of Latvia, and identification with all humanity as predictors of emigration plans (Figure 3). Participants' gender, age, graduation, employment, experience of studying or working abroad, and ethnolinguistic group were controlled to test effects revealed in the general population (Study 1). The model demonstrated a slightly lowered fit in incremental indices, $\chi_{\left(867\right)}^{2}$ = 1312.10, *p* < 0.001, CFI = 0.896, TLI = 0.883, RMSEA = 0.046 with 95% CI [0.041, 0.051], *p*_*RMSEA*_ = 0.919, and SRMR = 0.065. After excluding two nonsignificant predictors—employment and experience abroad—the model demonstrated an acceptable fit, $\chi_{\left(797\right)}^{2}$ = 1183.94, *p* < 0.001, CFI = 0.907, TLI = 0.895, RMSEA = 0.045 with 95% CI [0.040, 0.050], *p*_*RMSEA*_ = 0.963, and SRMR = 0.065. It confirmed the role of a general sense of country as the main predictor of considering emigration. The negative effect of *Spatiotemporal Commitment* to Latvia remained in power, while the positive effect of *Influence* was nonsignificant. Identification with the people of Latvia closely correlated with the sense of country and did not predict considering emigration. Identification with all humanity correlated negatively with the sense of country and positively predicted emigration plans.

Demographic variables also explained the concepts under investigation. Age positively predicted the sense of country and national identification, while it negatively predicted identification with all humanity. Latvian speakers displayed a higher level of the sense of country and identification with the people of Latvia. Graduate participants reported a heightened sense of country compared to nongraduates. All predictors together explained 52% of the variance of emigration plans. Removing the sense of country from the predictive model reduced the explained variance to 21%. In this case, identification with the people of Latvia demonstrated a significant negative relationship with planning emigration, β = −0.21, *p* = 0.041. In turn, excluding national identification resulted in 48% of the variance being explained, and the general factor of the sense of country remained the main predictor of emigration plans, β = −0.49, *p* < 0.001. These results confirmed the incremental validity of the sense of country compared to national identification in predicting emigration plans.

### Discussion

The results confirmed the structure of the sense of country in a group of predominantly studying adults. The relationship between the sense of country and national identification is very close, and the link between specific factors of *Relational Belonging* and *Bond* supports their conceptual concordance. The sense of country and spatiotemporal commitment predicted emigration plans negatively. IWAH related to the sense of country negatively and was a positive predictor of emigration plans.

The best model of relationships between the sense of country and national identification included two closely related general constructs. Additional linking of *Relational Belonging* and *Bond* revealed the closest elements of these general constructs. It concurs with the significance of bonds, solidarity, and belonging in social identification expressed by Leach et al. (2008). The level of correlation allows consideration of one specific factor interacting with the sense of country and national identification. Simultaneously, the sense of country is a broader psychological construct integrating components of person–environment interaction, as also demonstrated in studies on the sense of community (e.g., Lardier et al., 2022). Within the sense of country, the sense of belonging combines with perceived opportunities for personal goals and control over the processes in the country, reflecting the unity of the main processes of socialization (Nurmi, 2004). This integrative structure explains nonsignificant relationships between national identification and planning emigration in the presence of the sense of country, confirming the leading role of the sense of country in predicting emigration plans.

Effects of demographic variables predominantly confirmed findings in the general population. Higher education and belonging to the majority (Latvian speakers) positively predicted the sense of country. Current employment demonstrated no significant link to planning emigration. Relatively small effects of age and gender were not topical in the current sample. Simultaneously, the experience of studying or working abroad did not relate to the sense of country or considering emigration, in contrast to Study 1. Considering different types of mobility (Gustafson, 2009), the convenience sample can represent a greater level of academic mobility, which can be less associated with the decision to emigrate.

The prediction of considering emigration by the sense of country, IWAH, and national identification is in line with SIMIC views on identity pathways (Haslam et al., 2021). The identity continuity pathway expresses as a lower level of considering emigration among adults with a higher spatiotemporal commitment and a general sense of country, which links to a higher sense of national identification. The identity gain pathway is visible in negative relationships between the sense of country and IWAH and the positive role of IWAH in predicting emigration plans.

The results also confirmed the possibility of presenting IWAH within a bifactor model. Nonsignificant loadings of items on *Concern* indicate their total inclusion in the general factor. This predominantly concurs with the findings of Hamer et al. (2021) on the bifactor model, indicating that only one item—helping all humans—has a significant load on the specific factor. These results inform a discussion on the structure of global identification and the balance of factors in IWAH.

## General discussion

Both studies confirm that the sense of country combines multiple dimensions of the person–environment interaction at the national level. Based on the model of the sense of community (McMillan and Chavis, 1986), the sense of country involved perceived influence, perceived opportunities for self-related and others-related goals, relational belonging, and spatiotemporal commitment to the country. The established bifactor model emphasizes that the sense of country integrates these dimensions. These findings also confirm the unity of components of channeling, selection, adjustment, and reflection as the processes of socialization (Nurmi, 2004). The structure of perceived opportunities reflects individual interaction with fields of production and reproduction, including economic activity, founding a family, and parenting (Nurmi, 2004). Involved in channeling, selection, and adjustment, personal and collective agency (Bandura, 2002; Heckhausen and Buchmann, 2019) represent individual subjectness during socialization (Nurmi, 2004). Reflections on involvement and acceptance at the national level and linking personal life with the country present a sense of belonging (Hagerty et al., 1992; Baumeister and Leary, 1995) and continuity in time (Sani et al., 2008; David and Bar-Tal, 2009; Kolesovs, 2021).

The contribution of specific factors to emigration plans confirms the complexity of interacting constructs. Reflecting continuity of belonging (Kolesovs, 2021), spatiotemporal commitment demonstrated a double effect on emigration plans in both studies. The stability of this trend indicates that continuity, referring to a commitment to the country, can also involve lower levels of social systems (Kolesovs, 2019) and physical places (Arcidiacono et al., 2007; Wenham, 2020). Additionally, temporal commitment interacts with personal goals and anticipated future, forming an individual future orientation (Nurmi, 1991; Seginer et al., 2004; Andre et al., 2018). In turn, the specific effect of perceived influence on emigration plans was not presented in the sample from the academic environment. Therefore, the effect of agency is significant in a broader population, which has greater variability in age, employment, and other demographic variables.

Exploring the relationships between the sense of country and national and global identification provides evidence for both pathways, emphasized in SIMIC (Haslam et al., 2021). The general factor of the sense of country predicts emigration plans, spatiotemporal commitment adds to this effect, and relational belonging links the sense of country to national identification. These findings reflect the social identity continuity pathway in planning emigration. In turn, global identification relates to a weaker sense of country and more certain plans regarding emigration, confirming the relationship between a broader social identification and the social identity gain pathway. Therefore, planning emigration leads to exclusive rather than inclusive national and global identifications, and the search for a new identity and transcending national borders is associated with a lower level of the sense of country.

### Limitations

Undoubtedly, the study has several limitations. Focusing on the structure of the sense of country limits exploration of its practical applications. The current findings emphasize a need for multifocal work to strengthen the sense of country. Simultaneously, a negative role of permanent emigration does not mean that other types of mobility harm the country's development. Further studies should include a broader view of mobility activities. In addition, limited international mobility during the COVID-19 pandemic potentially impacted mobility plans. The study should be repeated now that this mobility has been restored.

Another group of limitations relates to sampling. The Study 1 sample closely resembles the characteristics of the population but overrepresents some segments (e.g., well-educated people). The sample of Study 2 also involves highly educated people which limits generalization to a broader population. Considering the pandemic-associated limitations of personal contact, a shift in characteristics is related to online questioning. A more favorable situation for personal communication can lead to correcting these disproportions.

One further limitation relates to the country. Latvia is small in territory and population, and the distance between community and country levels is potentially close. This means that the relationship between the sense of country and community is a topic for further cross-cultural comparison.

Two additional directions of study can compensate for the current deficiencies. First, measurement of social identifications occurred in the frame of IWAH. It involved two levels of social systems but limited measurement of other components of social identification. As a result, the findings overrepresent the dimension of self-investment, while self-definition (Leach et al., 2008) is not presented. Second, specific components of the sense of country are a topic for further investigation. For example, it seems valuable to differentiate the gradient of an expected change in commitment from the level of commitment to the country.

### Conclusions

The sense of country integrates a set of psychological constructs, reflecting individual socialization and the person–environment interaction at the national level. More pragmatic influence and perceived opportunities merge with spatiotemporal commitment and belonging to the country. The combined effects of the general and specific factors predicting emigration plans confirm the complexity of this interaction and point to connections of the sense of country to other levels of social systems and individual future orientation. Relational belonging closely links to national bonds, revealing the conceptual intersection of the sense of country and national identification. In addition, the relationships between the sense of country and national identification reflect social identity continuity, while global identification refers to its change when planning emigration.

## Data availability statement

The original contributions presented in the study are included in the article/Supplementary material, further inquiries can be directed to the corresponding author.

## Ethics statement

The studies involving human participants were reviewed and approved by Research Ethics Committee of the Institute of Cardiology and Regenerative Medicine of the University of Latvia. Written informed consent for participation was not required for this study in accordance with the national legislation and the institutional requirements.

## Author contributions

The author confirms being the sole contributor of this work and has approved it for publication.

## Funding

Study 1 was performed within the individual project supported by the University of Latvia (Grant No. Y5-AZ22-ZF-N-040). In scientific cooperation, the inventory was applied for data collection in the project supported by the National Research Program No. VPP-IZM-2018/1-0013. Study 2 was conducted as an extension of research Grant No. Y5-AZ22-ZF-N-040.

## Conflict of interest

The author declares that the research was conducted in the absence of any commercial or financial relationships that could be construed as a potential conflict of interest.

## Publisher's note

All claims expressed in this article are solely those of the authors and do not necessarily represent those of their affiliated organizations, or those of the publisher, the editors and the reviewers. Any product that may be evaluated in this article, or claim that may be made by its manufacturer, is not guaranteed or endorsed by the publisher.
